# Establishing a Framework for the Clinical Translation of Germline Findings in Precision Oncology

**DOI:** 10.1093/jncics/pkaa045

**Published:** 2020-05-29

**Authors:** Katherine Dixon, Sean Young, Yaoqing Shen, My Linh Thibodeau, Alexandra Fok, Erin Pleasance, Eric Zhao, Martin Jones, Geraldine Aubert, Linlea Armstrong, Alice Virani, Dean Regier, Karen Gelmon, Dan Renouf, Stephen Chia, Ian Bosdet, S Rod Rassekh, Rebecca J Deyell, Stephen Yip, Ana Fisic, Emma Titmuss, Shirin Abadi, Steven J M Jones, Sophie Sun, Aly Karsan, Marco Marra, Janessa Laskin, Howard Lim, Kasmintan A Schrader

**Affiliations:** p1 Department of Medical Genetics, University of British Columbia, Vancouver, British Columbia, Canada; p2 Department of Pathology and Laboratory Medicine, University of British Columbia, Vancouver, British Columbia, Canada; p3 Canada’s Michael Smith Genome Sciences Centre, BC Cancer, Vancouver, British Columbia, Canada; p4 Terry Fox Laboratory, BC Cancer Research Centre, Vancouver, British Columbia, Canada; p5 Provincial Medical Genetics Program, Children’s & Women’s Health Centre of British Columbia, Vancouver, British Columbia, Canada; p6 Ethics Service, Provincial Health Service of Authority of BC, Vancouver, British Columbia, Canada; p7 Canadian Centre for Applied Research in Cancer Control, Cancer Control Research, BC Cancer, Vancouver, British Columbia, Canada; p8 School of Population and Public Health, University of British Columbia, Vancouver, British Columbia, Canada; p9 Division of Medical Oncology, BC Cancer, Vancouver, British Columbia, Canada; p10 Cancer Genetics and Genomics Laboratory, BC Cancer, Vancouver, British Columbia, Canada; p11 BC Children’s Hospital Research Institute, Vancouver, British Columbia, Canada; p12 Division of Hematology/Oncology and BMT, Department of Pediatrics, University of British Columbia, Vancouver, British Columbia, Canada; p13 Department of Pharmacy, BC Cancer, Vancouver, British Columbia, Canada; p14 Hereditary Cancer Program, BC Cancer, Vancouver, British Columbia, Canada; p15 Department of Molecular Oncology, BC Cancer, Vancouver, British Columbia, Canada

## Abstract

Inherited genetic variation has important implications for cancer screening, early diagnosis, and disease prognosis. A role for germline variation has also been described in shaping the molecular landscape, immune response, microenvironment, and treatment response of individual tumors. However, there is a lack of consensus on the handling and analysis of germline information that extends beyond known or suspected cancer susceptibility in large-scale cancer genomics initiatives. As part of the Personalized OncoGenomics program in British Columbia, we performed whole-genome and transcriptome sequencing in paired tumor and normal tissues from advanced cancer patients to characterize the molecular tumor landscape and identify putative targets for therapy. Overall, our experience supports a multidisciplinary and integrative approach to germline data management. This includes a need for broader definitions and standardized recommendations regarding primary and secondary germline findings in precision oncology. Here, we propose a framework for identifying, evaluating, and returning germline variants of potential clinical significance that may have indications for health management beyond cancer risk reduction or prevention in patients and their families.

Characterizing hereditary genetic variation in high- and moderate-penetrance cancer predisposition genes may have implications for cascade carrier testing, cancer risk-reduction, and screening interventions in individuals at risk of having inherited a causal germline variant. Individual variability in disease prognosis and treatment response also occurs within the context of heterogeneous genetic backgrounds, including rare variants associated with cancer susceptibility and common polymorphisms in other cancer-related genes (1–4). However, standards for the clinical translation of genetic information relevant to cancer susceptibility, pathogenesis, prognosis, and treatment, as well as secondary or incidental genetic information unrelated to cancer, are inconsistent across cancer genomics programs (5). This may result from varying regional policies regarding the return of research results, clinician preference, or genetic literacy.

The Personalized OncoGenomics (POG) program is a precision medicine initiative in British Columbia (BC), Canada, that was established to identify clinically actionable molecular events in adult metastatic cancer patients and pediatric patients with poor prognosis cancers (NCT02155621). Whole-genome sequencing (WGS) and RNA sequencing of fresh, frozen tumor biopsies performed with WGS of paired normal tissues has helped identify somatic alterations and inherited genetic variants that shape tumor progression (6–9). This and similar projects, such as the Cancer Genome Atlas and International Cancer Genome Consortium, have provided important resources for understanding the morbid human genome (10, 11). At the same time, the rate of data production has far outpaced the development of evidence-based guidelines for managing germline findings. Here, we advocate for a broader understanding of what defines primary germline findings in oncology and propose a framework for identifying, evaluating, and reporting research germline findings within the clinical infrastructure of a publicly funded provincial health authority.

## Early Years of the POG Program

Since its establishment in 2012, the POG program has continued to develop effective, collaborative, and transparent data management and reporting strategies (6). These have allowed the accommodation of advancements in technology, computational pipelines, therapeutic developments, and biological and clinical knowledge. Before standard recommendations for variant interpretation were published by the American College of Medical Genetics and Genomics (ACMG) and Association for Molecular Pathology in 2015, germline data were assessed on an ad hoc basis to identify high-penetrance variants relevant to inherited cancer susceptibility or treatment (12). Any research findings were discussed with treating oncologists as part of the POG tumor board, but this approach was not broadly or consistently actionable in a maturing program. This was complicated by conflicting interpretations of variant pathogenicity and/or differing opinions regarding the return of results, indicating the need for a standardized procedure for germline assessment. Given the ethical challenges of germline data analysis within the oncology setting, the POG Ethics and Germline Working Group was created in 2014. Using autonomy, beneficence, nonmaleficence, and justice as guiding principles, the mandate of the Ethics and Germline Working Group includes addressing ongoing issues related to the management and clinical translation of germline findings. The group meets monthly and consists of a multidisciplinary team of oncologists, medical and molecular geneticists, pathologists, bioinformaticians and other scientists, an ethicist, and lawyers.

## Transparency and Standardization of Informed Consent

This POG program is approved by the University of British Columbia Research Ethics Committee, and written informed consent or assent is obtained for all patients involved in this research. The Ethics and Germline Working Group reached a consensus that informed consent or assent should include by default the reporting of any clinically actionable cancer-related germline genetic information for both adults and children with cancer. This mandate keeps with the primary goal of the POG program to characterize the complete genomic architecture of an individual cancer while maintaining data transparency and allowing opportunities for clinical translation. Germline findings of clinical significance related to cancer risk are currently returned to the research participant, designee, or next of kin by the patient’s oncologist, and a referral is made to the provincial BC Cancer Hereditary Cancer Program (HCP) for genetic counseling and clinical variant confirmation. For both tumor-only and tumor-normal sequencing, an appropriate consent procedure includes a detailed review of family history and pretest counseling regarding the potential risks and benefits of germline findings. This is critical for ensuring ethical justification of studies involving the analysis of hereditary genetic variation.

An opt-in procedure for the return of germline findings of clinical significance unrelated to cancer has been adopted by the POG program, consistent with recommendations based on patient and public preference studies (13). Although these findings are not routinely sought in cancer genomics analyses, 97.9% of participants enrolled in the program between 2012 and July 2019 (n* *=* *993) opted for the return of incidental germline findings unrelated to cancer. These observations suggest that the level of potential risk in learning about inherited genetic variation, including risks related to cancer susceptibility, disease carrier status, or paternity, is acceptable to patients and has not resulted in a decline in participation in the program. The potential for learning about genetic cancer predisposition in particular should be an essential part of the patients’ education and communicated to them at the time of diagnosis.

## Germline Variant Curation, Validation, and Return

**Figure 1.**
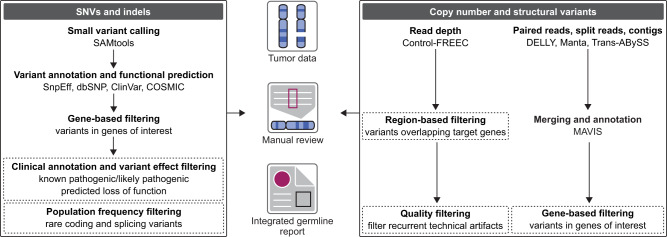
Approach for germline variant calling, annotation, and filtering in tumor-normal whole-genome sequencing. In the Personalized OncoGenomics program, parallel pipelines are implemented for the analysis of small variants, including single nucleotide variants (SNVs) and small insertions and deletions (indels), and structural variants (SVs). Low-complexity regions, strong GC bias, and repetitive elements limit the accuracy of SV calling through short-read (50-300 bp) sequencing. Consequently, complementary read depth-, flanking read-, split read-, and contig-based computational approaches are incorporated to increase the sensitivity of SV detection. COSMIC = Catalog of Somatic Mutations in Cancer; MAVIS = Merging, Annotation, Validation, and Illustration of Structural Variants. Germline variants with known or putative clinical significance are prioritized by clinical annotation, functional effect prediction, and population frequency in 98 cancer predisposition genes. All candidate variants are reviewed in a genome browser to flag possible technical artifacts, and this information is included in an integrated germline report along with relevant tumor data.

**Figure 2.**
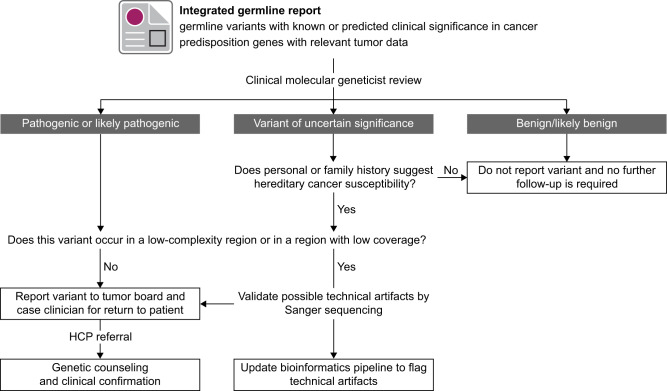
Standard procedure for the review, reporting, and clinical translation of germline variants in the Personalized OncoGenomics program. Given an integrated germline analysis report, a clinical molecular geneticist at the Cancer Genetics and Genomics Laboratory (CGL) curates all germline variants with known or potential clinical significance in cancer predisposition genes undergoing prioritized review. The Ethics and Germline Working Group determine, by consensus, final recommendations for variant reporting and whether a referral to the Hereditary Cancer Program (HCP) should be made for patient counseling and clinical genetic testing. In the absence of functional evidence supporting pathogenicity, variants of uncertain significance are disclosed only when the patient’s personal or family history is suggestive of hereditary cancer susceptibility. Clinically actionable variants that occur in areas of the reference genome flagged as low-complexity or repetitive regions will be validated at CGL prior to return of results. If false-positive variants are identified, an updated bioinformatics pipeline is implemented to flag these variants in future cases.

Pathogenic and likely pathogenic germline variants in moderate- to high-penetrance cancer predisposition genes underlie 5%-10% of all cancers, with a prevalence of up to 20% in certain cancer types (14, 15). In collaboration with HCP and the Cancer Genetics and Genomics Laboratory (CGL), the Ethics and Germline Working Group developed standard procedures for germline variant prioritization and evaluation (Figures 1 and 2). Genome-wide variant calling is performed in parallel pipelines for small variants, including single nucleotide variants and small insertions and deletions, and structural variants (SVs), including copy number variants and balanced genomic rearrangements (Figure 1). SV calling through short-read–based next-generation sequencing in particular has inherent technical and computational challenges because of low-complexity sequences in the human reference genome. Therefore, using multiple computational tools that employ complementary variant calling methods is preferred to improve the sensitivity of SV detection. Following gene- and function-based filtering, candidate germline variants are manually reviewed in a genome browser to flag putative technical or sequence artifacts prior to clinical review.

To allow rapid return of results for variants with established clinical actionability, a tiered list of cancer susceptibility genes is analyzed according to the following guidelines. Known pathogenic and likely pathogenic variants in curated variant databases, such as ClinVar and local CGL database, and novel predicted loss of function variants (eg, deletion, frameshift, nonsense, canonical splice site, and stop loss) are prioritized for curation in known cancer predisposition genes (Supplementary Table 1, available online). For several genes associated with high-penetrance syndromes, variants of uncertain significance (VUS) and rare variants in coding and splice regions are further prioritized for review. Variant classification is performed by a clinical molecular geneticist from CGL and certified by the Canadian College of Medical Genetics and Genomics or ACMG according to updated guidelines from the ACMG, Association for Molecular Pathology, and Clinical Genome Resource. This approach integrates clinical expertise and laboratory protocols in variant classification and confirmatory genetic testing, respectively, allowing seamless translation for patients referred to HCP (Figure 2). Final recommendations for return of information and referral for genetic counseling and clinical testing are made based on consensus among the Ethics and Germline Working Group. In exceptional cases, germline variants may be subject to expedited review by a core expert panel, including a clinical molecular geneticist, medical geneticist, and oncologist, to allow immediate return of information and clinical referral (Supplementary Table 2, available online). Clinical genetics expertise and group consultation are thus integral parts of germline assessment in the POG program to ensure reliability, consistency, and transparency.

Evolving variant curation guidelines indicate the need for dynamic computational pipelines that integrate updated variant information from population and clinical databases and encourage efficient retrospective analysis (16, 17). To aid in large-scale variant identification and classification, special consideration should also be given to variants with founder effects, specific modes of disease inheritance, and variants with low to moderate cancer risk. Founder mutations in common cancer predisposition genes such as *BRCA1*, *BRCA2*, *CHEK2*, and *MUTYH* should be excluded from global allele frequency thresholds used in automated variant filtering. For example, more than 1% of patients in the POG program are carriers for pathogenic *MUTYH* variants, reflecting a strong representation of individuals of East and Southeast Asian descent in BC (9, 18). In such cases, we recommend developing highly curated internal databases with known pathogenic and likely pathogenic germline variants to reduce the incidence of false-negative findings. Furthermore, variants in Mendelian disease genes underlying cancer predisposition syndromes inherited in autosomal recessive or X-linked recessive patterns, such as *MUTYH*-associated polyposis and dyskeratosis congenita, respectively, also require exceptional committee review. The position of the Ethics and Germline Working Group is to evaluate the risks and benefits of returning carrier status for autosomal recessive cancer susceptibility genes on a gene- and case-specific basis. Finally, identification of low- to moderate-penetrance cancer predisposition variants may present an additional challenge because of limited evidence-based guidelines regarding effective clinical management and cancer risk reduction strategies. Personal and family medical history should be carefully considered in these cases to determine the appropriateness of variant disclosure for cancer susceptibility.

For individuals with phenotypic indications of high-penetrance cancer predisposition syndromes and uninformative clinical genetic testing, evaluation of tumor WGS and RNA sequencing may improve genetic diagnosis through the resolution of potential splicing variants, noncoding variants in regulatory regions, or structural variants (9, 19). Tumor data may also inform possible roles for VUS or autosomal recessive gene variants in pathogenesis, recently demonstrated in a patient with biallelic variants in *MUTYH* (p.Gly286Glu and p.Ser346Ser) with tumor evidence supporting global deficiency in base excision repair (7–9). The specific types of molecular data that could be considered in whole-genome and transcriptome studies include genome-wide mutation burden, simple somatic mutations and mutational signatures, copy number alterations, loss of heterozygosity, structural variants and structural variant signatures, expression outliers, expression-based classifications, and alternative splicing (Supplementary Table 3, available online). However, recommendations for incorporating tumor data into variant classification are still evolving, and recent guidelines recommend cautious variant interpretation based on this evidence alone without corresponding data from in vitro or in vivo functional studies (20). In the POG program, VUS are not reclassified on the basis of tumor data alone without ClinVar classifications or published phenotypic and functional evidence supporting pathogenicity. For patients with relevant personal or family history, VUS may be returned to the treating clinician with a recommendation for referral to HCP if the individual is eligible for publicly funded index genetic testing. The Ethics and Germline Working Group does not support clinical decision making based on the presence of VUS alone and stresses the importance of consultation with core members of a clinical genetics team to ensure appropriate clinical follow-up when indicated.

## Implications for Germline Genetic Variation Beyond Cancer Susceptibility

**Figure 3.**
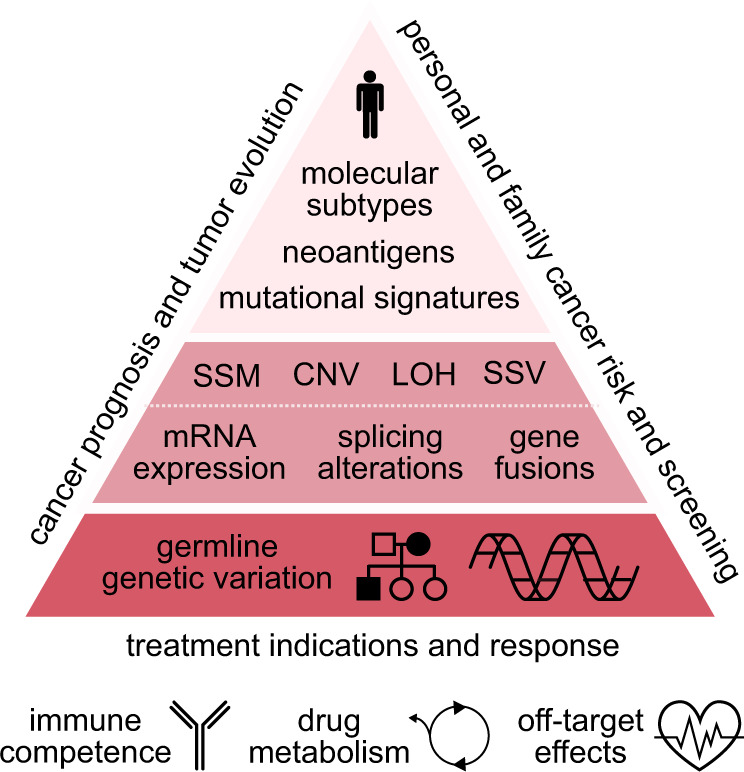
Extending the clinical significance of integrated molecular analysis of tumor and normal tissues beyond cancer susceptibility. CNV = copy number variants; LOH = loss of heterozygosity; mRNA = messenger RNA; SSM = simple somatic mutations; SSV = somatic structural variants.

Both germline variation and somatic alterations have potential clinical significance in precision oncology, and integrated analysis of tumor-normal sequencing may identify roles for germline variation that extend beyond cancer susceptibility. Therefore, our framework for germline data management defines primary germline findings as any variants with relevance to tumor biology or treatment. This includes variants with implications for estimating cancer risk, determining the landscape of somatic alterations or tumor evolution, modifying the immune response and microenvironment, and predicting overall response or adverse reactions to treatment (Figure 3). This broad definition is based on findings from large tumor-sequencing projects that have allowed functional characterization of inherited genetic variation other than rare coding variants in known disease genes. These include a number of recent studies that have identified roles for common and noncoding variants in tumorigenesis that may ultimately guide treatment interventions or development of targeted therapeutics (21–24). Identifying germline variation with potential tumor relevance is thus an important part of characterizing the complex molecular architecture of individual tumors and may have immediate or future clinical implications.

## Framework for the Clinical Translation of Germline Findings

Based on our experience during 7 years of the POG program, we developed an integrated schema for the management of germline genetic information. We propose a nonmutually exclusive categorical framework for evaluating clinically actionable germline variants that aims to identify inherited cancer susceptibility, characterize treatment indications, and provide opportunities for the discovery of novel genetic associations (Table 1). This includes pathogenic and likely pathogenic variants in known cancer predisposition genes with defined cancer risk estimates and screening recommendations and may include VUS in cases where the patient’s phenotype supports pathogenicity. In the POG program, known and novel pathogenic and likely pathogenic variants in known cancer predisposition genes are reviewed by the Ethics and Germline Working Group, and these are then disclosed to the tumor board and case oncologist with a general recommendation for return to the patient with a referral to HCP for follow-up. Secondary germline findings in the 59 genes defined by the ACMG should consistently be returned to patients if informed consent for return of such findings was given at the time of study enrollment (25, 26).

**Table 1.** Framework for the return of germline findings in precision oncology

Tier	Clinical indication	Recommended categories for consideration	Examples	PEWG recommendations for disclosure
Primary	Cancer susceptibility	Known or novel pathogenic and likely pathogenic variants associated with moderate- to high-penetrance cancer susceptibility^a^	*CHEK2* c.1100delC (p.Thr367Metfs)	Return to patient with referral to HCP
		Known or novel pathogenic and likely pathogenic variants associated with low-penetrance cancer susceptibility^a^	*CHEK2* c.470T>C (p.Ile157Thr)	Return to patient with referral to HCP on a case-by-case basis
		Autosomal or X-linked carrier status for known or novel pathogenic and likely pathogenic variants associated with low-, moderate- or high-penetrance cancer susceptibility^a^	*ERCC5* c.529-1G>A	Return to patient with referral to HCP on a case-by-case basis
		VUS in cancer predisposition genes with clinical and molecular indications for pathogenicity	*MUTYH* c.996G>A (p.Ser332Ser)	Return to patient with referral to HCP on a case-by-case basis
	Disease pathogenesis, prognosis, or treatment	Known or novel variants in cancer predisposition genes understood to contribute to tumor phenotype and evolution	Biallelic loss through combined germline and somatic aberrations: *NTHL1* carrier status *MUTYH* carrier status	Return to patient with referral to HCP on a case-by-case basis and if clinical significance has been established
		Deleterious variants in known or novel cancer-related genes	Deletion polymorphisms in *APOBEC3A* and *APOBEC3B*	Return to patient if clinical significance has been established
		Pharmacogenomic variants with established or potential cancer treatment associations	UGT1A1*28	Return to patient if clinical significance has been established
		Alleles associated with immune response	HLA class I genotypes	Return to patient if clinical significance has been established
		Variants in gene(s) reviewed at the request of the case clinician given patient consent	*SCN5A* c.1673A>G (p.His558Arg)	Return to patient if clinical significance has been established
Secondary	Mendelian disease risk or carrier status	Deleterious variants in genes without known implications for cancer prevention, cancer screening, or treatment	Pathogenic and likely pathogenic variants in Mendelian disease genes defined by the ACMG	Return to patient with referral to medical genetics program if patient preference for noncancer-related information is indicated in consent

^a^ Implications for cancer susceptibility should be considered in the context of variant zygosity and the typical mode of inheritance observed for a given gene. In particular, this includes variants in autosomal recessive genes or in X-linked genes conferring recessive disease risk that may have differing indications for XY, XX, and XO individuals. ACMG = American College of Medical Genetics and Genomics; HCP = Hereditary Cancer Program; HLA = human leukocyte antigen; PEWG = Personalized OncoGenomics Ethics and Germline Working Group; VUS = variants of uncertain significance.

With an inclusive definition of primary germline findings in the context of cancer genomics, pharmacogenomic variants with known drug associations, polymorphic alleles with putative immune response associations, variants in genes with potential tumor relevance based on histological or molecular characteristics, and other variants with potential clinical relevance as requested by the case oncologist could also be analyzed. The Ethics and Germline Working Group supports routine reporting of germline variants predictive of immune response, such as human leukocyte antigen class I genotypes, but recommends caution in reporting pharmacogenomic variants with limited or conflicting scientific evidence that may prevent use of important supportive medications (27). Clinicians and scientists should refer to public curated databases such as PharmGKB for updated variant reviews and clinical guidelines (28). In practice, a curated variant database relevant for chemotherapy and other cancer therapy with established pharmacogenomic associations, such as gastrointestinal and bone marrow toxicity in carriers of the UGT1A1*28polymorphism, should be prioritized during routine germline analysis unless otherwise requested by the case clinician (29).

Pharmacogenomic variants will be an important aspect of cancer treatment in the era of precision medicine. The potential benefits of profiling pharmacogenomic variants as part of the POG program were demonstrated in a patient with a gastrointestinal stromal tumor and prolonged QT interval during a high-dose course of imatinib. Further genomic profiling was performed at the request of the case clinician, and a common polymorphism in *SCN5A* (p.His558Arg) that has been previously reported as a genetic modifier in cardiac arrhythmia syndromes was identified (30, 31). Metabolic and cardiovascular gene variants that are known to modulate drug metabolism or mediate adverse events in response to treatment have immediate clinical utility in treatment selection. Thus, prior knowledge of variants that can impact treatment choice may provide indications for or against the use of certain drugs. In the case described previously, this may have indicated an avoidance of specific tyrosine kinase inhibitors associated with prolonged QT intervals (32, 33).

Novel relationships between genetic alterations and histological or molecular phenotypes could also be investigated through an agnostic analysis of germline variation. Mutation and expression comparisons using public datasets are invaluable tools for characterizing the individual tumor genome and in validating putative molecular associations. However, these must be interpreted cautiously given expected differences between sample handling and preparation protocols, sequencing chemistries and platforms, and computational pipelines. With a secondary goal of discovery, deleterious germline variants in known cancer genes defined in the Cancer Gene Census of the Catalog of Somatic Mutations in Cancer or genes in other disease-related pathways could be prioritized but not limiting when investigating novel associations with outlier tumor phenotypes (Supplementary Table 4, available online) (34, 35). It should be noted that germline findings with unconfirmed implications for disease pathogenesis, prognosis, or treatment are purely the result of research and, because of their uncertain nature, are strongly discouraged from being returned to the patient unless indicated by the tumor board for treatment indications. If germline findings are determined to be of clinical importance, notice should be given to the case clinician to ensure banking of a clinical DNA sample and patient and/or family referral for genetic counseling and variant confirmation. Disclosure of germline variants to the POG tumor board and case clinician is encouraged in cases with established implications for clinical management, including for cancer risk reduction, screening, or treatment indications. In the future, larger studies and agnostic analyses may uncover additional roles for these variants in disease pathogenesis that may be predictive or prognostic of overall survival and treatment outcomes.

Hereditary cancer susceptibility is commonly observed in unselected cohorts of cancer patients who do not meet current clinical testing guidelines (36, 37). In precision oncology, the utility of exploring inherited genetic variation is achieved through the implementation of cancer prevention and screening strategies and by the use of targeted therapies. However, personnel and financial resources in jurisdictions with universal health care may be a growing barrier to service accessibility as genetic testing becomes more common and additional germline variants with clinical significance are discovered. These issues were strongly considered by the Ethics and Germline Working Group in assessing the benefits of disclosure for certain variants, but these guidelines must be re-evaluated moving forward to help inform cost-effective, patient-centered care. Based on our experience in the POG program, genome-wide analysis of inherited genetic variation should allow for the examination of normal and disease-causing variation that may affect tumor evolution, response to therapy, and immune function; predict adverse events; and/or be relevant to noncancer disease risk that may be meaningful to patients and their care.

## Funding

This work was supported by Genome Canada and Genome BC (202SEQ, 212SEQ, 12002), Canada Foundation for Innovation (20070, 30198, 30981, 33408), and the BC Knowledge Development Fund. We gratefully acknowledge the participation of our patients and families, the POG team, and the generous support of the BC Cancer Foundation and Genome British Columbia (B20POG). MLT is supported by the University of British Columbia Clinician Investigator Program. KAS is supported by the Canadian Institutes of Health Research and the Michael Smith Foundation for Health Research.

## Notes

**Role of the funder:** The funders had no role in the writing of the manuscript or the decision to submit the manuscript for publication.

**Disclosures:** GA is a part-time employee of Repeat Diagnostics, a company providing clinical telomere length measurements services. All other authors have no conflicts of interest.

**Acknowledgments:** This work would not be possible without the participation of our patients and families, the POG team, and the generous support of the BC Cancer Foundation and Genome British Columbia. We would also like to thank Jessica Nelson, Katherine Mui, Lindsay Zibrik, Kevin Sauve, and Kirstin Brown for program support, data collection, and editing assistance.

**Author contributions:** KD and KAS prepared the manuscript and conceptualized the framework based on POG Germline and Ethics Working Group discussions. SY, IB, and AK provided clinical molecular genetics expertise. YS, MLT, EP, EZ, MJ, ET and SJMJ were involved in data analysis and bioinformatics methods development. A. Fok provided program management support. GA provided gene-specific molecular genetics expertise. LA, KG, D. Renouf, SC, SRR, RJD, SY, A. Fisic, SS, JL, HL, and KAS provided clinical expertise and were involved in patient care. AV, D. Regier and SA provided expertise in ethics, economics and public health, and pharmacogenomics, respectively. HL chairs the POG Germline and Ethics Working Group. MM, JL, HL and KAS conceptualized this work. All authors reviewed and edited the manuscript.

## Supplementary Material

pkaa045_Supplementary_Data
