# Supplementary material for: Establishing a Framework for the Clinical Translation of Germline Findings in Precision Oncology
Source: JNCI Cancer Spectr. 2020 May 29;4(5):pkaa045. doi: 10.1093/jncics/pkaa045 (PMC7583151; doi:10.1093/jncics/pkaa045)
Supplement: pkaa045_Supplementary_Data [file pkaa045_supplementary_data.pdf]

Supplementary Materials

Establishing a framework for the clinical translation of germline findings in precision oncology

Katherine Dixon, MSc, Sean Young, PhD, Yaoqing Shen, PhD, My Linh Thibodeau, MD, MSc, Alexandra Fok, MSc, Erin Pleasance, PhD, Eric Zhao, PhD, Martin Jones, PhD, Geraldine Aubert, PhD, Linlea Armstrong, MD, Alice Virani, PhD, Dean Regier, PhD, Karen Gelmon, MD, Dan Renouf, MD, Stephen Chia, MD, Ian Bosdet, PhD, S. Rod Rassekh, MD, Rebecca J. Deyell, MD, Stephen Yip, MD, PhD, Ana Fisic, BScN, Emma Titmuss, MSc, Shirin Abadi, PharmD, MBA, Steven J.M. Jones, PhD, Sophie Sun, MD, Aly Karsan, MD, Marco Marra*, PhD, Janessa Laskin*, MD, Howard Lim*, MD, Kasmintan A. Schrader*, MBBS, PhD

*Co-senior authors

Table S1. Bioinformatics tools currently used for germline analysis in the Personalized OncoGenomics program.

Analysis Step	Software	Version	PMID
Read alignment	BWA	0.5.7-0.7.6	20080505
Small variant calling	samtools	0.1.17	19505943
Small variant annotation	SnpEff	4.1	22728672
Copy number calling	Control-FREEC	N/A	22155870
Structural variant calling	DELLY	0.7.3	22962449
	ABySS	1.3.4	19251739
	manta	1.0.0	26647377
Structural variant consolidation and annotation	MAVIS	N/A	30016509

Table S2. Cancer predisposition genes regularly reviewed for candidate pathogenic and likely pathogenic germline variants in the Personalized OncoGenomics program.

Gene	Entrez	OMIM	Inheritance	Locus	CGL	ACMG	Genotype-Phenotype Relationships
ABRAXAS1 (FAM175A)	84142	611143	AD	4q21.23			Candidate breast cancer susceptibility gene that is clinically available
AKT1	207	164730	SM	14q32.33			Breast cancer, somatic [114480]; Colorectal cancer, somatic [114500]; Cowden syndrome 6, [615109]; Ovarian cancer, somatic [167000]; Proteus syndrome, somatic [176920]
ALK	238	105590	AD	2p23.2			Neuroblastoma, susceptibility to [613014]
APC	324	611731	AD	5q21-q22	*	*	Adenoma, periampullary, somatic; Adenomatous polyposis coli [175100]; Brain tumor-polyposis syndrome 2 [175100]; Colorectal cancer, somatic [114500]; Desmoid disease, hereditary [135290]; Gardner syndrome [175100]; Gastric cancer, somatic [613659]; Hepatoblastoma, somatic [114550]
ATM	472	607585	CX	11q22.3			Ataxia-telangiectasia [208900]; Lymphoma, B-cell non-Hodgkin, somatic; Lymphoma, mantle cell; T-cell prolymphocytic leukemia, somatic; Breast cancer, susceptibility to [114480]
ATR	545	601215	AR	3q22-q24			Cutaneous telangiectasia and cancer syndrome, familial [614564]; Seckel syndrome 1 [210600]
AXIN2	8313	604025	AD	17q24			Colorectal cancer, somatic [114500]; Oligodontia-colorectal cancer syndrome [608615]
BAP1	8314	603089	AD	3p21.1			Tumor predisposition syndrome [614327]
BARD1	580	601593	AD	2q34-q35			Breast cancer, susceptibility to [114480]
BLM	641	604610	AR	15q26.1			Bloom syndrome [210900]
BMPR1A	657	601299	AD	10q22.3	*	*	Juvenile polyposis syndrome, infantile form [174900]; Polyposis syndrome, hereditary mixed, 2 [610069]; Polyposis, juvenile intestinal [174900]
BRCA1	672	113705	AD	17q21	*	*	Breast-ovarian cancer, familial, 1 [604370]; Pancreatic cancer, susceptibility to, 4 [614320]
BRCA2	675	600185	CX	13q12.3	*	*	Fanconi anemia, complementation group D1 [605724]; Pancreatic cancer [613347]; Prostate cancer [176807]; Wilms tumor [194070]; Breast cancer, male, susceptibility to [114480]; Breast-ovarian cancer, familial, 2 [612555]; Glioblastoma 3 [613029]; Medulloblastoma [155255]; Pre-B-cell acute lymphoblastic leukemia
BRIP1	83990	605882	AD	17q22			Breast cancer, early-onset [114480]; Fanconi anemia, complementation group J [609054]
CBL	867	165360	AD	11q23.3			Noonan syndrome-like disorder with or without juvenile myelomonocytic leukemia [613563]
CDC73	79577	607393	AD	1q25-q31			Hyperparathyroidism, familial primary [145000]; Hyperparathyroidism-jaw tumor syndrome [145001]; Parathyroid adenoma with cystic changes [145001]; Parathyroid carcinoma [608266]

CDH1	999	192090	AD	16q22.1	*	Endometrial carcinoma, somatic [608089]; Gastric cancer, familial diffuse, with or without cleft lip and/or palate [137215]; Ovarian carcinoma, somatic [167000]; Breast cancer, lobular [114480]; Prostate cancer, susceptibility to [176807]
CDK4	1019	123829	AD	12q14		Melanoma, cutaneous malignant, 3 [609048]
CDKN1B	1027	600778	AD	12p13		Multiple endocrine neoplasia, type IV [610755]
CDKN2A	1029	600160	AD	9p21		Melanoma and neural system tumor syndrome [155755]; Orolaryngeal cancer, multiple; Pancreatic cancer melanoma syndrome [606719]; Melanoma, cutaneous malignant, 2 [155601]
CHEK2	11200	604373	AD	22q12.1		Li-Fraumeni syndrome [609265]; Osteosarcoma, somatic [259500]; Breast and colorectal cancer, susceptibility to; Breast cancer, susceptibility to [114480]; Prostate cancer, familial, susceptibility to [176807]
DICER1	23405	606241	AD	14q32.13		Goiter, multinodular 1, with or without Sertoli-Leydig cell tumors [138800]; Pleuropulmonary blastoma [601200]; Rhabdomyosarcoma, embryonal, 2, [180295]
DKC1	1736	305000	XLR	Xq28		Dyskeratosis congenita, X-linked
EGFR	1956	131550	AD	7p11.2		Adenocarcinoma of lung, response to tyrosine kinase inhibitor in [211980]; Nonsmall cell lung cancer, response to tyrosine kinase inhibitor in [211980]; Nonsmall cell lung cancer, susceptibility to [211980]
EPCAM	4072	185535	CX	2p21		Colorectal cancer, hereditary nonpolyposis, type 8 [613244]; Diarrhea 5, with tufting enteropathy, congenital [613217]
ERCC2	2068	126340	AR	19q13.2-q13.3		Cerebrooculofacioskeletal syndrome 2 [610756]; Trichothiodystrophy [601675]; Xeroderma pigmentosum, group D [278730]
ERCC3	2071	133510	AR	2q21		Trichothiodystrophy [601675]; Xeroderma pigmentosum, group B [610651]
ERCC4	2072	133520	AR	16p13.3-p13.13		Fanconi anemia, complementation group Q [615272]; XFE progeroid syndrome [610965]; Xeroderma pigmentosum, group F [278760]; Xeroderma pigmentosum, type F/Cockayne syndrome [278760]
ERCC5	2073	133530	AR	13q33		Xeroderma pigmentosum, group G [278780]; Xeroderma pigmentosum, group G/Cockayne syndrome [278780]
ETV6	2120	616216	AD	12p13.2		Thrombocytopenia 5
EZH2	2146	601573	AD	7q36.1		Weaver syndrome [277590]
FANCA	2175	607139	AR	16q24.3		Fanconi anemia, complementation group A [227650]
FANCC	2176	613899	AR	9q22.3		Fanconi anemia, complementation group C [227645]
FH	2271	136850	CX	1q42.1		Fumarase deficiency [606812]; Leiomyomatosis and renal cell cancer [150800]
FLCN	201163	607273	AD	17p11.2		Birt-Hogg-Dube syndrome [135150]; Colorectal cancer, somatic [114500]; Pneumothorax, primary spontaneous [173600]; Renal carcinoma, chromophobe, somatic [144700]

GATA2	2624	137295	AD	3q21.3			Dendritic cell, monocyte, B lymphocyte, and natural killer lymphocyte deficiency [614172]; Emberger syndrome [614038]; Leukemia, acute myeloid, susceptibility to [601626]; Myelodysplastic syndrome, susceptibility to [614286]
GREM1	26585	603054	AD	15q13.3			Hereditary mixed polyposis syndrome
HNF1A	6927	142410	AD	12q24.31			Diabetes mellitus, insulin-dependent, 20 [612520]; Hepatic adenoma, somatic [142330]; MODY, type III [600496]; Renal cell carcinoma [144700]; Diabetes mellitus, insulin-dependent [222100]; Diabetes mellitus, noninsulin-dependent, 2 [125853]
HRAS	3265	190020	AD	11p15.5			Congenital myopathy with excess of muscle spindles [218040]; Costello syndrome [218040]; Schimmelpenning-Feuerstein-Mims syndrome, somatic mosaic [163200]; Bladder cancer, somatic [109800]; Nevus sebaceous, somatic [162900]; Thyroid carcinoma, follicular, somatic [188470]
IDH1	3417	147700	SM	2q34			Glioma, susceptibility to, somatic [137800]
KIT	3815	164920	AD	4q12			Gastrointestinal stromal tumor, familial [606764]; Germ cell tumors [273300]; Leukemia, acute myeloid [601626]; Mast cell disease [154800]; Piebaldism [172800]
MAX	4149	154950	AD	14q23.3			Pheochromocytoma, susceptibility to [171300]
MEN1	4221	613733	AD	11q13.1		*	Adrenal adenoma, somatic; Angiofibroma, somatic; Carcinoid tumor of lung; Lipoma, somatic; Multiple endocrine neoplasia 1 [131100]; Parathyroid adenoma, somatic
MET	4233	164860	AD	7q31.2			Hepatocellular carcinoma, childhood type [114550]; Renal cell carcinoma, papillary, 1, familial and somatic [605074]
MITF	4286	156845	AD	3p14-p13			Tietz albinism-deafness syndrome [103500]; Waardenburg syndrome, type 2A [193510]; Waardenburg syndromeocular albinism, digenic [103470]; Melanoma, cutaneous malignant, susceptibility to, 8 [614456]
MLH1	4292	120436	AD	3p22.2	*	*	Colorectal cancer, hereditary nonpolyposis, type 2 [609310]; Mismatch repair cancer syndrome [276300]; Muir-Torre syndrome [158320]
MRE11	4361	600814	CX	11q21			Ataxia-telangiectasia-like disorder [604391]
MSH2	4436	609309	AD	2p21	*	*	Colorectal cancer, hereditary nonpolyposis, type 1 [120435]; Mismatch repair cancer syndrome [276300]; Muir-Torre syndrome [158320]
MSH6	2956	600678	AD	2p16.3	*	*	Colorectal cancer, hereditary nonpolyposis, type 5 [614350]; Endometrial cancer, familial [608089]; Mismatch repair cancer syndrome [276300]
MUTYH	4595	604933	AR	1p34.1	*	*	Adenomas, multiple colorectal [608456]; Colorectal adenomatous polyposis, autosomal recessive, with pilomatricomas [132600]; Gastric cancer, somatic [613659]
NBN	4683	602667	CX	8q21.3			Aplastic anemia [609135]; Leukemia, acute lymphoblastic [613065]; Nijmegen breakage syndrome [251260]
NF1	4763	613113	AD	17q11.2			Leukemia, juvenile myelomonocytic [607785]; Neurofibromatosis, familial spinal [162210]; Neurofibromatosis, type 1 [162200]; Neurofibromatosis-Noonan syndrome [601321]; Watson syndrome [193520]

NF2	4771	607379	AD	22q12.2		*	Meningioma, NF2-related, somatic [607174]; Neurofibromatosis, type 2 [101000]; Schwannomatosis [162091]
NSD1	64324	606681	AD	5q35.2-q35.3			Beckwith-Wiedemann syndrome [130650]; Sotos syndrome 1 [117550]
PALB2	79728	610355	CX	16p12.2	*		Fanconi anemia, complementation group N [610832]; Breast cancer, susceptibility to [114480]; Pancreatic cancer, susceptibility to, 3 [613348]
PAX5	5079	167414	AD	9p13.2			Leukemia, acute lymphoblastic, susceptibility to, 3 [615545]
PDGFRA	5156	173490	AD	4q12			Gastrointestinal stromal tumor, somatic [606764]; Hypereosinophilic syndrome, idiopathic, resistant to imatinib [607685]
PHOX2B	8929	603851	AD	4p13			Central hypoventilation syndrome, congenital, with or without Hirschsprung disease [209880]; Neuroblastoma with Hirschsprung disease [613013]; Neuroblastoma, susceptibility to, 2 [613013]
PIK3CA	5290	171834	SM	3q26.32			Breast cancer, somatic [114480]; CLOVE syndrome, somatic [612918]; Colorectal cancer, somatic [114500]; Cowden syndrome 5 [615108]; Gastric cancer, somatic [613659]; Hepatocellular carcinoma, somatic [114550]; Keratosis, seborrheic, somatic [182000]; Megalencephaly-capillary malformation-polymicrogyria syndrome, somatic [602501]; Megalencephaly-polymicrogyria-polydactyly-hydrocephalus syndrome, somatic [603387]; Nevus, epidermal, somatic [162900]; Nonsmall cell lung cancer, somatic [211980]; Ovarian cancer, somatic [167000]
PMS1	5378	600258	AD	2q31-q33			Note: PMS1 was initially identified as a candidate gene for Lynch Syndrome, but further evidence was unsupportive. Here, it is included in regular analysis as it is tested on some germline colon cancer susceptibility sequencing panels.
PMS2	5395	600259	AD	7p22.1	*	*	Colorectal cancer, hereditary nonpolyposis, type 4 [614337]; Mismatch repair cancer syndrome [276300]
POLD1	5424	612591	AD	19q13.33	*		Colorectal cancer, susceptibility to, 10
POLE	5426	174762	AD	12q24.33	*		FILS syndrome [615139]; Colorectal cancer, susceptibility to, 12 [615083]
PRKAR1A	5573	188830	AD	17q24.2			Acrodysostosis 1, with or without hormone resistance [101800]; Adrenocortical tumor, somatic; Carney complex, type 1 [160980]; Myxoma, intracardiac [255960]; Pigmented nodular adrenocortical disease, primary, 1 [610489]; Thyroid carcinoma, papillary, somatic [188550]
PTCH1	5727	601309	AD	9q22.32			Basal cell carcinoma, somatic [605462]; Basal cell nevus syndrome [109400]; Holoprosencephaly-7 [610828]
PTEN	5728	601728	AD	10q23.31	*	*	Bannayan-Riley-Ruvalcaba syndrome [153480]; Cowden syndrome 1 [158350]; Endometrial carcinoma, somatic [608089]; Lhermitte-Duclos syndrome [158350]; Macrocephalyautism syndrome [605309]; Malignant melanoma, somatic [155600]; PTEN hamartoma tumor syndrome; Squamous cell carcinoma, head and neck, somatic [275355]; Thyroid carcinoma, follicular, somatic [188470]; VATER association with macrocephaly and ventriculomegaly [276950]; Glioma susceptibility 2 [613028]; Meningioma [607174]; Prostate cancer, somatic [176807]

PTPN11	5781	176876	AD	12q24.13			LEOPARD syndrome 1 [151100]; Leukemia, juvenile myelomonocytic [607785]; Metachondromatosis [156250]; Noonan syndrome 1 [163950]
RAD50	10111	604040	CX	5q31.1			Nijmegen breakage syndrome-like disorder [613078]
RAD51	5888	179617	AD	15q15.1			Mirror movements 2 [614508]; Breast cancer, susceptibility to [114480]
RAD51B	5890	602948	AD	14q24.1			Breast cancer, susceptibility to [114480]
RAD51C	5889	602774	AD	17q22			Fanconi anemia, complementation group O [613390]; Breast-ovarian cancer, familial, susceptibility to, 3 [613399]
RAD51D	5892	602954	AD	17q12			Breast-ovarian cancer, familial, susceptibility to, 4 [614291]
RB1	5925	614041	AD	13q14.2		*	Bladder cancer, somatic [109800]; Osteosarcoma, somatic [259500]; Retinoblastoma [180200]; Retinoblastoma, trilateral [180200]; Small cell cancer of the lung, somatic [182280]
RECQL4	9401	603780	CX	8q24.3			Baller-Gerold syndrome [218600]; RAPADILINO syndrome [266280]; Rothmund-Thomson syndrome [268400]
RET	5979	164761	AD	10q11.21		*	Central hypoventilation syndrome, congenital [209880]; Medullary thyroid carcinoma [155240]; Multiple endocrine neoplasia IIA [171400]; Multiple endocrine neoplasia IIB [162300]; Pheochromocytoma [171300]; Renal agenesis [191830]; Hirschsprung disease, susceptibility to, 1 [142623]
RUNX1	861	151385	AD	21q22.12			Leukemia, acute myeloid [601626]; Platelet disorder, familial, with associated myeloid malignancy [601399]
SDHA	6389	600857	CX	5p15.33			Cardiomyopathy, dilated, 1GG [613642]; Leigh syndrome [256000]; Mitochondrial respiratory chain complex II deficiency [252011]; Paragangliomas 5 [614165]
SDHAF2	54949	613019	AD	11q12.2		*	Paragangliomas 2 [601650]
SDHB	6390	185470	AD	1p36.13		*	Cowden syndrome 2 [612359]; Gastrointestinal stromal tumor [606764]; Paraganglioma and gastric stromal sarcoma [606864]; Paragangliomas 4 [115310]; Pheochromocytoma [171300]
SDHC	6391	602413	AD	1q23.3		*	Gastrointestinal stromal tumor [606764]; Paraganglioma and gastric stromal sarcoma [606864]; Paragangliomas 3 [605373]
SDHD	6392	602690	AD	11q23.1		*	Carcinoid tumors, intestinal [114900]; Cowden syndrome 3 [615106]; Merkel cell carcinoma, somatic; Paraganglioma and gastric stromal sarcoma [606864]; Paragangliomas 1, with or without deafness [168000]; Pheochromocytoma [171300]
SH2D1A	4068	300490	XR	Xq25			Lymphoproliferative syndrome, X-linked, 1 [308240]
SMAD4	4089	600993	AD	18q21.2	*	*	Juvenile polyposis hereditary hemorrhagic telangiectasia syndrome [175050]; Myhre syndrome [139210]; Pancreatic cancer, somatic [260350]; Polyposis, juvenile intestinal [174900]
SMARCA4	6597	603254	AD	19p13.2			Mental retardation, autosomal dominant 16 [614609]; Rhabdoid tumor predisposition syndrome 2 [613325]
SMARCB1	6598	601607	AD	22q11.23			Mental retardation, autosomal dominant 15 [614608]; Rhabdoid predisposition syndrome 1 [609322]; Rhabdoid tumors, somatic [609322]

STK11	6794	602216	AD	19p13.3	*	*	Melanoma, malignant, somatic; Pancreatic cancer [260350]; Peutz-Jeghers syndrome [175200]; Testicular tumor, somatic [273300]
SUFU	51684	607035	AD	10q24.32			Medulloblastoma, desmoplastic [155255]; Meningioma, familial, susceptibility to [607174]
TERC	7012	127550	AD	3q26.2			Dyskeratosis congenita, autosomal dominant 1
TERT	7015	187270	AD	5p15.33			Bone marrow failure, telomere-related, 1 [614742]; Coronary artery disease; Dyskeratosis congenita, autosomal dominant 2 [613989]; Dyskeratosis congenita, autosomal recessive 4 [613989]; Leukemia, acute myeloid [601626]; Melanoma, cutaneous malignant, 9 [615134]; Pulmonary fibrosis, telomere-related, 1 [614742]
TGFBR1	7046	190181	AD	9q22.33		*	Loeys-Dietz syndrome, type 1A [609192]; Loeys-Dietz syndrome, type 2A [608967]; Multiple self-healing squamous epithelioma, susceptibility to [132800]
TINF2	26277	613990	AD	14q12			Dyskeratosis congenita, autosomal dominant 3
TMEM127	55654	613403	AD	2q11.2			Pheochromocytoma, susceptibility to [171300]
TP53	7157	191170	AD	17p13.1	*	*	Adrenal cortical carcinoma [202300]; Breast cancer [114480]; Choroid plexus papilloma [260500]; Colorectal cancer [114500]; Hepatocellular carcinoma [114550]; Li-Fraumeni syndrome [151623]; Nasopharyngeal carcinoma [607107]; Osteosarcoma [259500]; Pancreatic cancer [260350]; Basal cell carcinoma 7 [614740]; Glioma susceptibility 1 [137800]
TSC1	7248	605284	AD	9q34.13		*	Focal cortical dysplasia, Taylor balloon cell type [607341]; Lymphangioleiomyomatosis [606690]; Tuberous sclerosis-1 [191100]
TSC2	7249	191092	AD	16p13.3		*	Lymphangioleiomyomatosis, somatic [606690]; Tuberous sclerosis-2 [613254]
VHL	7428	608537	CX	3p25.3		*	Erythrocytosis, familial, 2 [263400]; Hemangioblastoma, cerebellar, somatic; Pheochromocytoma [171300]; Renal cell carcinoma, somatic [144700]; von Hippel-Lindau syndrome [193300]
WRN	7486	277700	AR	8p12			Werner syndrome [277700]
WT1	7490	607102	CX	11p13		*	Denys-Drash syndrome [194080]; Frasier syndrome [136680]; Meacham syndrome [608978]; Mesothelioma, somatic [156240]; Nephrotic syndrome, type 4 [256370]; Wilms tumor, type 1 [194070]

OMIM: Online Mendelian Inheritance in Man; CGL: Cancer Genetics and Genomics Laboratory, BC Cancer; ACMG: American College of Medical Genetics and Genomics; Genotype-Phenotype Relationships: OMIM Phenotype [Phenotype MIM number].

Table S3. Sample report for expedited review of pathogenic and likely pathogenic germline variants in the Personalized OncoGenomics program.

Germline Variant Information		
"NBN has a germline mutation: I171V. This study suggests this variant is a risk factor for several malignancies: leukemia, melanoma, breast, ovarian, etc. https://www.ncbi.nlm.nih.gov/pmc/articles/PMC4665302/ NBN is in the homologous recombination pathway: https://www.ncbi.nlm.nih.gov/pmc/articles/PMC3944197/ BRCA1 has a germline VUS, L246V, but this study suggests that it's neutral: https://www.ncbi.nlm.nih.gov/pmc/articles/PMC2687711/ This patient has a strong BRCA1/2 deficiency signature and a moderately high HRD score. However, the clinician suggested that this is resistant to chemo (platinum?). PARP inhibitors may be an option for this patient? "		
If any of these boxes indicate "NO" or not filled in prior to the POG meeting, the variant should not be returned and should go to the monthly POG Ethics Working Group (PEWG) meeting.		
**Team**	**Indication**	**Response**
**Bioinformatics**		
Bioinformatician	Has the variant report been completed?	Yes
	Has the PEWG on-call team been informed of the variant?	Yes
**PEWG on-call**		
Primary oncologist<br>Clinical molecular geneticist<br>Medical geneticist<br>Project manager	Is there a representative oncologist on the team?	Yes
	Is there a clinical molecular geneticist on the team?	Yes
	Is there a medical geneticist on the team?	Yes
	Is there a POG project manager on the team?	Yes
	Has CGL determined that this is a presumed pathogenic variant in a known cancer predisposition gene or ACMG gene and can be confirmed through a clinical test?	No
	Is the PEWG on-call team unanimous in their support for returning this variant?	No
	Is the variant understood to be technically valid and pathogenic or likely pathogenic according to ACMG/AMP 2015 guidelines?	Uncertain
	Has the patient consented to the return of incidental findings in the case the variant is not related to cancer susceptibility?	Yes
	Has the primary oncologist been informed of the findings and referral process prior to the POG Tumour Board meeting? Note that a referral to HCP should be made regardless of whether patient has previously been referred for other indications.	pending
**Clinical**		
Primary oncologist	Primary care physician initiates referral to HCP using standard form with variant finding and preliminary POG germline report, if available at the time of referral.	pending
**PEWG**		
PEWG	If the variant is unresolved by the PEWG on-call team, variant review should proceed as normal.	pending

Table S4. Sample information from the Personalized OncoGenomics program's germline report. Two sections of the report, germline variants and gene- and/or pathway-related tumour data, are included to facilitate the evaluation of variant pathogenicity in disease pathogenesis.

Report Section	Description	Value
Germline variant information	Chromosome	13
	Position	32936732
	dbSNP ID	rs80359013
	Reference	G
	Alternate	C
	Quality	225
	Genotype	heterozygous
	Gene Symbol	*BRCA2*
	Transcript ID	NM_000059
	Transcript Biotype	protein_coding
	Functional Impact	missense
	HGVS cDNA	c.7878G>C
	HGVS Protein	p.Trp2626Cys
	gnomAD AF	7.96e-6 (2/251276)
	Flags[a]	ACMG, CGL
	ClinVar Significance	Pathogenic
	CGL Significance	Pathogenic
Tumour WGS and RNA-seq	Tumour Genotype	heterozygous
	Tumour WGS AD	0.44 (27/61)
	Tumour RNA-seq AD	0.2 (3/15)
	Simple Somatic Mutation	none
	Somatic CNV	none
	Loss of Heterozygosity	none
	Somatic SV	none
	RNA-seq RPKM	1.48
	RNA-seq GTEx %ile	100
	RNA-seq Normal TCGA %ile	100
	RNA-seq Tumour TCGA %ile	100
	RNA-seq AS	none
	RNA-seq Fusion	none
	Mutational Signature Outliers	Signature 3: 0.275<br>Signature 8: 0.244<br>Signature 1: 0.201

[a]Flags or warnings may indicate variants with known clinical interpretations of pathogenicity, founder variants, variants ACMG genes, variants in autosomal recessive or X-linked genes and other information obtained from internal databases.

AD: allelic depth; CNV: copy number variant; SV: structural variant; RPKM: reads per kilobase per million; %ile: percentile as compared to tissue-matched gene expression database; AS: alternative splicing
